# Classic Ketogenic Diet and Modified Atkins Diet in SLC2A1 Positive and Negative Patients with Suspected GLUT1 Deficiency Syndrome: A Single Center Analysis of 18 Cases

**DOI:** 10.3390/nu13030840

**Published:** 2021-03-04

**Authors:** Jana Ruiz Herrero, Elvira Cañedo Villarroya, Luis González Gutiérrez-Solana, Beatriz García Alcolea, Begoña Gómez Fernández, Laura Andrea Puerta Macfarland, Consuelo Pedrón-Giner

**Affiliations:** 1 Department of Pediatric Gastroenterology, Pediatric Service, San Rafael Hospital, 28016 Madrid, Spain; 2 Department of Gastroenterology and Nutrition, University Children’s Hospital Niño Jesús, 28009 Madrid, Spain; elviracaedo@yahoo.es (E.C.V.); bgarciaa@salud.madrid.org (B.G.A.); bgomezf@salud.madrid.org (B.G.F.); laura.puertamac@yahoo.es (L.A.P.M.); consuelocarmen.pedron@salud.madrid.org (C.P.-G.); 3 Department of Neurology, University Children’s Hospital Niño Jesús, 28009 Madrid, Spain; luisggsolana@hotmail.com; 4 Center for Biomedical Network Research on Rare Diseases (CIBERER), Health Institute Carlos III, 28029 Madrid, Spain

**Keywords:** GLUT1 deficiency syndrome, SLC2A1 gene, ketogenic diet, pediatric epilepsy, refractory epilepsy, movement disorder

## Abstract

Background: Glucose transporter type 1 deficiency syndrome (GLUT1DS) is caused by mutations in the SLC2A1 gene and produces seizures, neurodevelopmental impairment, and movement disorders. Ketogenic dietary therapies (KDT) are the gold standard treatment. Similar symptoms may appear in SLC2A1 negative patients. The purpose is to evaluate the effectiveness of KDT in children with GLUT1DS suspected SLC2A1 (+) and (-), side effects (SE), and the impact on patients nutritional status. Methods: An observational descriptive study was conducted to describe 18 children (January 2009–August 2020). SLC2A1 analysis, seizures, movement disorder, anti-epileptic drugs (AEDS), anthropometry, SE, and laboratory assessment were monitored baseline and at 3, 6, 12, and 24 months after the onset of KDT. Results: 6/18 were SLC2A1(+) and 13/18 had seizures. In these groups, the age for debut of symptoms was higher. The mean time from debut to KDT onset was higher in SLC2A1(+). The modified Atkins diet (MAD) was used in 12 (5 SLC2A1(+)). Movement disorder improved (4/5), and a reduction in seizures >50% compared to baseline was achieved in more than half of the epileptic children throughout the follow-up. No differences in effectiveness were found according to the type of KDT. Early SE occurred in 33%. Long-term SE occurred in 10, 5, 7, and 5 children throughout the follow-up. The most frequent SE were constipation, hypercalciuria, and hyperlipidaemia. No differences in growth were found according to the SLC2A1 mutation or type of KDT. Conclusions: CKD and MAD were effective for SLC2A1 positive and negative patients in our cohort. SE were frequent, but mild. Permanent monitoring should be made to identify SE and nutritional deficits.

## 1. Introduction

The main fuel for the human brain is glucose, and Glut-1 is the major glucose transporter expressed on the blood-brain barrier. The glucose transporter type 1 deficiency syndrome (GLUT1DS) was first described by De Vivo in 1991 [1] and consists of an inborn metabolic disease caused by the absence or loss of function of the Glut-1 protein coded in the SLC2A1 gene on chromosome 1 [2]. Hypoglycorrhachia in the setting of normoglycemia and low-to-normal cerebrospinal fluid lactate is the main biochemical disturbance. The classic phenotype consists in a developmental encephalopathy with seizures (usually refractory epilepsy), acquired microcephaly, and movement disorders. Atypical phenotypes without seizures have been described in recent years (i.e., complex movement disorders or paroxysmal episodes triggered by fasting or exercise) [3,4]. In addition, 5–10% of patients suffer similar symptoms and biochemical disturbances without the SLC2A1 gene mutation [5]. In these patients, an extended genetic testing may discover disturbances in other genes.

The current gold standard treatment for GLUT1DS are ketogenic dietary therapies (KDT), especially the classic ketogenic diet (CKD) [6]. CKD is a high fat and low carbohydrate diet that provides ketones as an alternative fuel to the brain. In the CKD, 87–90% of daily caloric intake comes from fat sources [7]. The provision of carbohydrates and proteins vary within ratios of 3:1 to 4:1 (3–4 g of fat to 1 g of carbohydrates plus protein). CKD reduces seizures and movement disorders, and it should be used lifelong [4]. However, side effects are common, and compliance is difficult. Other types of KDT have been used. The modified Atkins diet (MAD) allows an unrestricted amount of protein and fat, but does restrict carbohydrate intake to 10 g/day. The compliance is easier, and it has fewer side effects. MAD has been used for refractory epilepsy with good results. The use of MAD in GLUT1DS has been published recently showing promising results [8,9,10].

The aim of this paper was to assess the efficacy and side effects of KDT for the treatment of SLC2A1 positive and negative children with suspected GLUT1DS, and to establish the differences between MAD and CKD in efficiency, adverse effects, and nutritional status.

## 2. Materials and Methods

Data of all pediatric patients (<18 years old) who had been prescribed a KDT to treat a confirmed or suspected GLUT1DS in a tertiary care level hospital between January 2009 and August 2020 were collected. A GLUT1DS was suspected in patients with seizures or movement disorders associated with hypoglycorrhachia. The diagnosis was confirmed when SLC2A1 mutations were found.

It is a retrospective and prospective (since May 2015) observational descriptive study approved by the Committee for Ethics in Clinical Research of the University Children´s Hospital Niño Jesús (project identification code R-0002/15, date of approval 25 February 2015). Patients or their relatives were informed of the aims of the research and signed an informed consent. All data were collected anonymously. Full medical histories were reviewed to collect epidemiological data, symptoms, genetic disorders, and treatments used before the diet´s implementation.

CKD was indicated for patients younger than 3 years or with severe epilepsy. MAD was selected for children older than 3 years. Both types of KDT were introduced slowly, increasing the ketogenic ratio progressively, adjusting according to the patient’s tolerance and the appearance of adverse effects, without fasting or liquid restriction. We started KDT as an inpatient regime when a CKD was selected, the patient was <2 years, or the child did not live in proximity to medical care. The hospital admission provided more time to teach the caregivers, monitor ketosis, and manage side effects. We started KDT as an outpatient when a MAD was selected, the patient was >2 years and the family had been previously trained in diet management and could easily access the hospital in case of side effects. Nutrition expert nurses designed the diets and trained the caregivers in meal preparation and management of side effects. The recommendations of the World Health Organization (WHO) based on age and weight were used to estimate energy and protein requirements. Protein intake was calculated according to daily protein recommendations (generally 1 g per kilo per day in children over 1 year and 1.5 in children under 1). We used the Holliday and Segar method [11] to estimate minimum fluids requirements, although we always prescribed at least the WHO recommendation or more to decrease some adverse effects of the diet such as constipation, acidosis, dehydration, or hypercalciuria. CKD was started as a 1:1 ratio. The next day, the ratio was increased to 2:1, and the next day to 3:1. If tolerance was not good or adverse effects appeared, the ratio was lowered again and then we tried to reach the 3:1 ratio more slowly. Infants younger than 6 months were on a liquid diet based on Ketocal^®^. Older infants and children were on a diet based on natural foods supplemented with a fat emulsion consisting of MCT (Liquigen^®^ or MCT Oil Nutricia^®^) or with powders or liquids rich in lipids and low in carbohydrates, based on whey protein supplemented with amino acids and micronutrients (Ketocal^®^). For the onset of MAD, the reduction of carbohydrates in the diet was first recommended. The caregivers were then given a list of foods indicating how many grams of carbohydrates they contained, the amount of carbohydrates the patient had to eat each day, and sample menus. In addition, they were recommended to supplement the diet with specific formulas (Ketocal^®^) to achieve a 0.8–1.1 ratio. Caregivers had written information about the menus, the glycemia and ketonemia controls that they should carry out, the management of adverse effects, and the drugs that they could use in case of common illness in childhood such as fever or respiratory infections and that they do not affect ketosis. Families could also make telephone inquiries in case of doubts. The caregivers were instructed to perform urine ketone tests every 8 h during the first days of the diet. Glycemia and beta-hydroxybutyrate were also monitored using test strips. These controls were carried out once or twice a day during the first weeks. Once a stable situation was reached, controls were carried out 3–4 times a week. The frequency of the controls was increased when the patients suffered secondary effects or associated diseases.

All patients underwent a thorough examination before KDT and then after the 1st, 3rd, 6th, 12th month, and then yearly. In each medical examination, possible side effects were accounted for. Weight, height, head circumference, and body mass index (BMI) were analyzed. Parameters were adjusted by sex and age according to the growth charts of the WHO (except head circumference in children older than 2 years, for whom the Spanish charts were used). Laboratory assessment included complete blood count, biochemical and lipid profiles, gasometry, ions, urinary sediment and calcium, protein, and citrate/creatinine ratios, prealbumin and retinol binding protein (RBP), vitamins A, E, D, B12, B9, zinc, selenium, carnitine, and parathormone (PTH).

Two groups were compared according to the SLC2A1 mutation (SLC2A1 (+) or (-)-group) and according to symptoms (the epilepsy group or movement disorder group). The effectiveness was measured by the reduction in number of seizure (100% (seizure freedom), 90–100% improvement, 50–90%, <50%, 0% (no improvement), or an increase in the number of seizures) and anti-epileptic drugs (AEDS) in the epilepsy group, and by a reduction in the intensity and frequency of movement disorder.

Variables were registered in an Excel program table. All statistical analyses were carried out with SPSS version 16.0. Wilcoxon signed-rank test was used to compare the z-score values for weight, height, and BMI, and blood and urine markers, at baseline and throughout the follow-up intervals. The Mann–Whitney test was used to compare the effects of the different KDT on blood and urine markers. Fisher’s exact test was used to compare the efficiency according to the type of KDT, and two groups were compared based on the existence of the SLC2A1 mutations and type of symptoms (seizures versus movement disorder).

## 3. Results

The characteristics of the 18 patients recruited are shown in Table 1. SLC2A1 mutations (SLC2A1(+)) were found in six patients (33.3%), five had seizures, and one had exercise-induced paroxysmal dyskinesia. Two patients had de novo deletions on chromosome 1 (130 Kb and 1.69 Mb, respectively) that affected the SLC2A1 gene. A heterozygous pathogenic variant in SLC2A1 was identified in three children. One was de novo mutation c.388G > A (p.Gly130Ser). In the other two patients, the same mutation was found in relatives: c.823G > A (p.Ala275Thr) was found in an asymptomatic brother and in the asthenic mother; and c.1232A > G (p.Ans411Ser) was identified in the asymptomatic mother. In one patient who was followed in the neurology department of another center, the specific mutation of the SLC2A1 gene was not registered in the medical record. Twelve patients were SLC2A1(-). Disturbances in other genes were found in six, and 4/6 had epilepsy. A one-year-old girl started with myoclonic seizures related to the mutation c.2813G > A (p.Arg938His) in GRIN2B (glutamate ionotropic receptor NMDA type subunit 2B). Alterations in GRIN2B may cause intellectual disability, epilepsy, autism, and sometimes microcephaly, movement disorder, cortical visual impairment, and occasionally cortical developmental malformation. A boy had a neonatal epileptic encephalopathy with generalized tonic-clonic seizures associated with the heterozygous mutation c.619C > T (p.Arg207Trp) in KCNQ2 (potassium voltage-gated channel subfamily Q member 2). Mutations in KCNQ2 can cause neonatal seizures to severe neonatal epileptic encephalopathy. A two-year-old girl began with absences related to the de novo mutation c.T277delGC (p.Ala93Glyfs*113) in SLC6A1 (Solute carrier family 6 (neurotransmitter transporter, GABA), member 1). SLC6A1 mutations may produce cognitive impairment and epilepsy. The most common seizures are absences, myoclonic, and atonic seizures. GRIN2B-, KCNQ2-, and SLC6A1-related disorders are inherited in an autosomal dominant manner. A one-year-old boy debuted with focal epilepsy due to a de novo mutation in chromosome Xq26.3, which led to disturbances in SLC9A6 (solute carrier family 9 member A6) associated with Christianson syndrome (intellectual disability, epilepsy, ataxia, and postnatal microcephaly). The remaining two children were a girl with the de novo mutation c.965T > C (Ile322Thr) in NALCN (sodium leak channel, non-selective) associated with CLIFAHDD syndrome (congenital contractures of the limbs and face with hypotonia and developmental delay syndrome) who debuted with a severe intellectual disability, dystonia, and ataxia at the age of one; and a boy who had a complex movement disorder with a predominance of intentional and postural tremor and had the heterozygous pathogenic variant 619C > T (p.Arg207Trp) in NKX2-1 (NK2 homeobox 1). NKX2-1-disorders are associated with choreoathetosis and hypothyroidism.

Median age at debut was lower in the SLC2A1(+)-group (1.7 ± 1.7 versus 2.2 ± 2.2 years), and in the epilepsy group (1.8 ± 1.5 versus 2.7 ± 3.2 years). The mean of cerebrospinal fluid to the blood glucose ratio was 0.45 (0.24–0.61). This ratio was lower in the SLCA1(+)-group (0.39 ± 0.1 versus 0.49 ± 0.09). These differences were not statistically significant.

Almost one third of the children did not have seizures (Table 1), and 13 had developmental delay or a cognitive disability. The mean of baseline seizures in the epilepsy group was 3/day, and 9/13 children had seizures daily. The mean of AEDS used in the epilepsy group was 3.8 (range 1–8), and 12/13 children with seizures had been treated with at least 2 AEDS before the onset of KDT. The most often tested AEDS before KDT were: Valproic acid (12/13), levetiracetam (8/13), clobazam (6/13), and lamotrigine (6/13). Just before starting the diet, only three patients were taking only one AEDS. One patient was on a KDT previously, but it was withdrawn due to the side effects and loss of efficiency. Three patients did not improve with other treatments (coenzyme Q, vitamin C, and riboflavin; vitamin B6 and adrenocorticotropic hormone; vitamin B6).

The characteristics of KDT are shown in Table 1. Although the differences were not statistically significant, the median age at the onset of KDT was higher in SLC2A1(+)-group (8.8 versus 4.9 years), and in the epilepsy group (4.5 versus 3.5 years). The median time from clinic debut to the onset of KDT was longer in the SLC2A1(+)-group (7.1 versus 2.7 years) (*p* = 0.039). All diets were administered orally. MAD was used in 5/6 children SLC2A1(+) and 7/12 SLC2A1(-). All CKD were started in children younger than 3 years, except one SLC2A1 (-)-girl with CLIFAHDD syndrome who started a CKD in another hospital. All CKD were onset as an inpatient setting, and most MAD (8/12) began as an outpatient setting. One MAD was initiated in another center. Median age at the onset of KDT was 2 years and 3 months in the CKD group and 8 years and 3 months in the MAD group. In 3 SLC2A1(-)-patients, KDT were modified to improve compliance: 2 CKD switched to MAD (13 days and 17 months later), and 1 MAD switched to a low glycemic index treatment (after 19 months).

Ketosis (>2.4 µmol/L) was reached in a mean of 3.8 days. No significant differences were found depending on the type of diet or the gene mutation in the time needed to reach ketosis. However, beta-hydroxybutyrate levels were higher in patients following a CKD at 3 months (*p* = 0.05).

**Figure 1:**
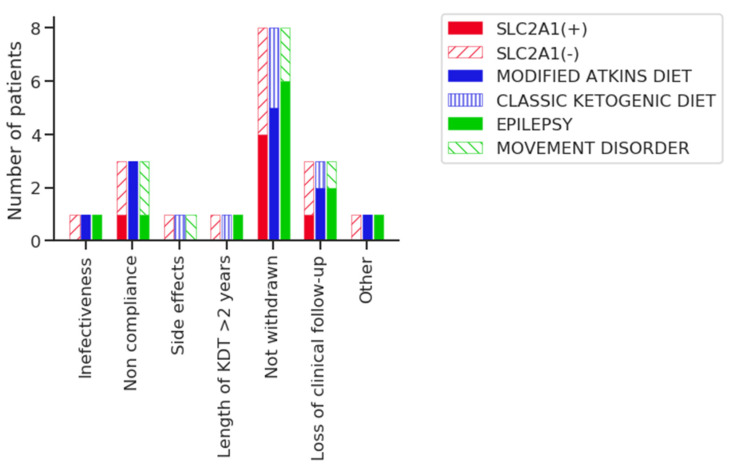
Reasons for withdrawing ketogenic dietary therapies (KDT) according to the SLC2A1 gene mutation, type of KDT, and symptoms.

Eight patients are still on a KDT (44.4%). Reasons for withdrawal are in Table 1 and Figure 1.

### 3.1. Outcome of the KDT

**Figure 2:**
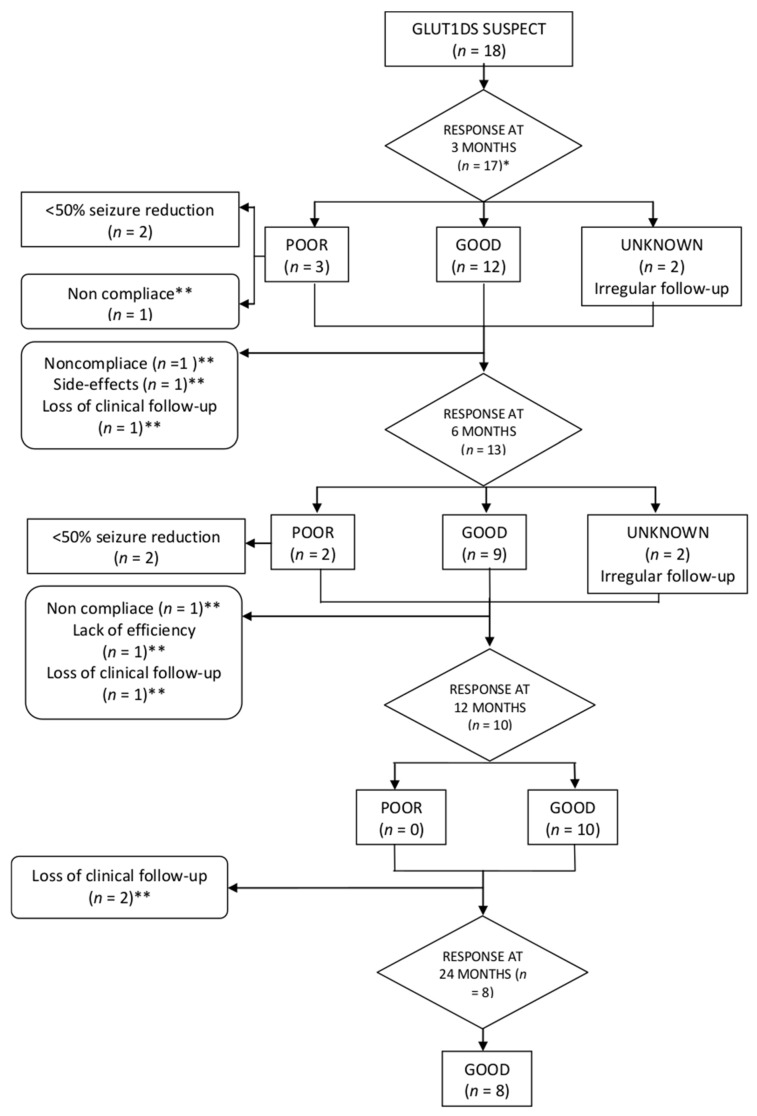
Evolution of the patients of the study. * One patient had not completed 3 months at the moment of the analysis. ** KDT withdrawn.

**Figure 3:**
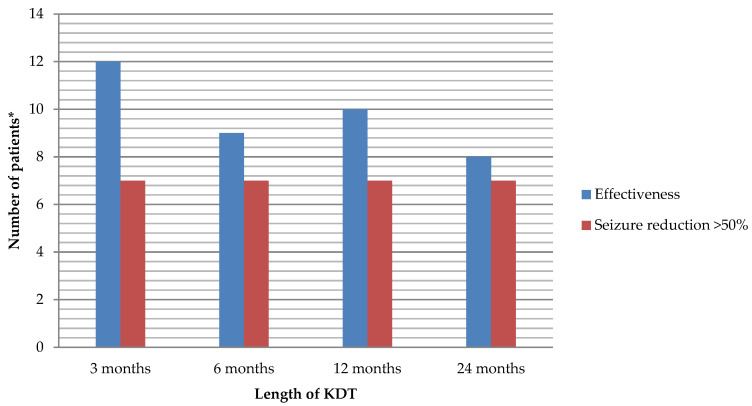
Global effectiveness and significant seizure reduction of the ketogenic dietary therapies (KDT) in patients with confirmed or suspected glucose transporter type 1 deficiency syndrome (GLUT1DS). * Each column represents the total number of patients without distinguishing the type of diet. Total number of patients on KDT: 17 at 3 months, 13 at 6 months, 10 at 12 months, and 8 at 24 months.

Figure 2 represents the flow chart with the evolution of the patients of the study. Patients who responded to the KDT are shown in Figure 3.

In epilepsy group, seizure-free patients were 6, 5, 6, and 7, and subjects with a reduction in the number of AEDS were 3, 4, 3 and 2 at 3, 6, 12, and 24 months, respectively.

The mean of the AEDS taken by the patients was 1.9 (±0.8) at 3 months, 1.5 (±0.7) at 6 months, 1.4 (±0.9) at 12 months, and 1.3 (±1.2) at 24 months. Throughout the follow-up, 4, 5, 5, and 6 patients, at 3, 6, 12, and 24 months, respectively, were taking ≤1 AEDS. Seven patients with epilepsy were on a KDT for more than 2 years (3 SLC2A1(+)), all of them had 90–100% improvement, and 3 of them were on a KDT for 6 years or more.

In the movement disorder group (*n* = 5), 4 children had a good response at 3 months. At 6 months, 2 patients were still on KDT (2 had the diet withdrawn, one due to non-compliance and one due to a high level of transaminases and the negative result of SLC2A1). Only one patient with a movement disorder due to the CLIFAHDD syndrome was still on a CKD, 6 years after the onset.

No significant differences were found in either in the reduction of seizures and AEDS, nor in the improvement of the movement disorder according to the type of KDT or SLC2A1 mutation.

### 3.2. Side Effects

Six children (33.3%) had early side effects (first month): Hypoglycemia with nausea/vomiting; asymptomatic hypoglycemia; drowsiness and hypercholesterolemia; carnitine deficiency; constipation; and metabolic acidosis and hypercalciuria. Long-term adverse effects are shown in Table 2. A higher risk of adverse effects was not found according to the type of diet, but significant differences were found in some blood and urine markers. The most clinically important were found in cholesterol (higher in patients on MAD at 3 months (*p* = 0.045)) and calciuria (higher at 3 months in patients on a CKD (*p* = 0.011)). Only one girl on a CKD with CLIFAHDD syndrome had nephrocalcinosis at 24 months.

### 3.3. Nutritional Evolution

One patient was severely malnourished and two were baseline overweight. BMI was normal in most patients throughout the follow-up. Only one patient was severely malnourished at 12 months, and one child was overweight, and one was mildly malnourished at 24 months. Mild disturbances in height were found in one child baseline, and in 2 at 6 months, and 3 at 12 and 24 months. Baseline mean z-score (SD) of BMI and height were −0.26 (1.6) and −0.63 (1.1), respectively. Throughout the follow-up, mean z-score (±SD) of BMI was: −0.20 (0.93), −0.30 (0.99), −0.30 (1.5), and 0.03 (1.4). Height data were: −0.65 (0.64), −0.70 (1.08), −1.2 (1.6), and −1.3 (1.5) at 3, 6, 12, and 24 months. No significant differences were found in anthropometric parameters throughout the follow-up compared to baseline, nor by the presence of the SLC2A1 gene mutation or the type of KDT.

Nutritional markers and micronutrients are shown in Table 3. At the baseline, the following nutrients were deficient: Vitamin D (3 patients), selenium (2), phosphorus (2), folic acid (1), and vitamin B12 (1). Throughout the follow-up, only two children had a vitamin A deficiency, one patient had a vitamin E deficit, and another one a vitamin D deficit. No significant differences were found in nutritional markers in blood except in phosphorus levels, which were significantly lower at 3 months (*p* = 0.007) and at 12 months (*p* = 0.028), compared to baseline.

Calcium at 3 months (*p* = 0.01), and prealbumin (*p* = 0.05) and folic acid (*p* = 0.025) at 12 months, were significantly higher in patients on CKD.

## 4. Discussion

GLUT1DS is a rare metabolic disease and its clinical presentation is heterogeneous [3,12,13]. Among our patients, epilepsy was the most frequent symptom. Various types of seizures were described, as in other studies [14,15], but the most frequent were myoclonic seizures and absences. Almost a third of the patients in our study had a motor disorder. Dystonia and paroxysmal movements were the most frequent movement disorders in our cohort. In one case, paroxysmal movements were paroxysmal exertion-induced dystonia. A high clinical suspicion is necessary for diagnosis since early treatment is important, because those patients who began KDT at a younger age had better outcomes [16,17]. When GLUT1DS is suspected, the SLC2A1 gene should be tested. However, some patients with compatible symptoms and a good response to treatment do not have mutations in SLC2A1 [2,18]. We used KDT in all our patients despite not having a definitive diagnosis because they had hypoglycorrhachia. Furthermore, the presence of a motor disorder increased the chances of being a GLUT1DS. On the other hand, almost all children with seizures had refractory epilepsy, and KDT should be considered when two AEDS have failed. In our cohort, only six patients with GLUT1DS suspected were finally confirmed, but most children benefited from KDT. The time from debut to the onset of the diet was greater than 3 years. This time was estimated to be more than 8 years in some papers [13,17]. The age at the onset of symptoms was lower in our patients with epilepsy and in the SLC2A1 (+)-group, and the time from debut to the onset of treatment was significantly higher. Most studies describe a delay of at least 4 years from debut to the onset of the diet [13,17,19]. Therefore, KDT should be onset when a GLUT1DS is suspected, although the definitive diagnosis is not made.

The current standard treatment for GLUT1DS are the KDT, especially the CKD [4,6,12,19,20]. Recent reviews have reported that 60% of the patients were seizure-free after the implementation of the diet, and 80% of patients with movement disorders improved [3,13]. In our cohort, 31–54% of patients with epilepsy were seizure-free, and 80% of patients with a movement disorder improved.

Although CKD is the most accepted treatment, two-thirds of our patients were treated with MAD, and no significant differences in efficiency were found according to the type of KDT. Several case reports describe the use of MAD [8,9,21,22], and Amalou and coworkers [10] evaluated the effectiveness of MAD in 10 patients with GLUT1DS: Epilepsy improved in all patients, and movement disorders also enhanced. Fujii et al. [23] presented the outcome of 39 patients with GLUT1DS. MAD and CKD were used respectively in 17 (55%) and 11 patients (35%), and both were effective on seizures and movement disorders. MAD may be as effective as CKD for the treatment of confirmed and suspected GLUT1DS according to our results and those of the authors mentioned [10,23].

When a GLUT1DS is confirmed, KDT should be used life-long. However, CKD is a very restricted diet, and non-compliance is a frequent reason for withdrawal, as reported a while ago [13] and recent [19] referring figures of 18%. Considering that MAD may be as effective as CKD, and that MAD is less restrictive, the type of KDT chosen should be individualized [24]. Curiously, the three patients who abandoned the diet due to difficulties in compliance in our study were on a MAD. A possible reason that justifies difficulties in compliance of MAD may be the age of the patients, since the mean age at the onset of MAD was higher. CKD is usually indicated for young children in whom it is easier to control the diet since it is based on liquid formulas or mashed foods. However, as the child grows, the child’s tastes can cause difficult compliance. All children who withdrew the diet in our study due to non-compliance were older than 3 years. Another cause that can lead to poorer compliance is the non-confirmation of GLUT1DS, which occurred in 2 of the children.

The adverse effects of KDT are quite common. Digestive [25,26], hepatic [26], and kidney problems [27,28,29] can appear, but are generally mild and easy to manage. In our cohort CKD seems to be as safe as MAD. Dyslipidemia [30,31] and kidney stones [27,28,29] are some of the most relevant long-term side effects. Hypercholesterolemia was very frequent in our cohort, but no patients were treated with lipid-lowering drugs, and, although CKD has a higher fat content diet, cholesterol was significantly higher in children who were on a MAD in the first few months. Dyslipidemia and cardiovascular risk have been studied. Short-term effects (after 12 weeks) on body fat distribution in 10 children affected by GLUT1DS, showed no alteration in the abdominal fat distribution [32]. Long-term, Heussinger and co-authors [33] followed 10 children and observed that the initial dyslipidemia may be transient, and they concluded that KDT do not increase long-term cardiovascular risk. Hypercalciuria was also common, but only one case had nephrocalcinosis in our cohort. All children with hypercalciuria were treated with oral citrates that prevent kidney stone formation [29], but may lead to bone mineral loss. It should be noted that urinary calcium/creatinine ratio was higher in patients on a CKD. Therefore, permanent monitoring of urine calcium/creatinine levels should be done in all patients on a KDT, but it is especially important in those on a CKD.

Deficits have been described in plasma levels of selenium, magnesium, carnitine, and vitamin D in patients with refractory epilepsy and KDT [34,35,36], but few studies have been performed in patients with GLUT1DS [37]. All our children took a carbohydrate-free multivitamin and mineral supplement from the beginning of the diet, as established in current guidelines [24]. Mild deficiencies in fat-soluble vitamins were found, and low phosphorus levels appeared in some patients. Frequent monitoring of nutritional markers and micronutrients in blood was important to detect these alterations early and prescribe other supplements when necessary.

The effects of KDT on growth have been widely studied, but it remains controversial. Several studies have shown that children on a KDT for more than 6 months decreased their growth rate [38,39,40,41,42], especially younger children [39]. Recent studies showed that 20–30% of children on KDT could have growth retardation [43,44,45]. The decrease in growth may be reversed after patients are taken off the KDT [46], but KDT in GLUT1DS should be used life-long. Long-term treatment with KDT can significantly affect height, especially in those children who start the diet before the age of 2 years [47]. Ferraris and co-authors [43] retrospectively investigated the occurrence of linear growth retardation in 34 children on a KDT, 20 of them with GLUT1DS, of which 16 did not show growth retardation at 12 months. A prospective study was designed by Armeno et al. [48] to evaluate growth in 45 children on a KDT. They observed growth deceleration in 9%. In our study we did not find significant differences in the z-score of weight, height, and BMI according to the presence of the SLC2A1 mutation, nor in the baseline or throughout the follow-up. No significant differences were found in those anthropometric parameters after the onset of KDT compared to baseline, and according to the type of KDT. However, our cohort is small, and the number of patients treated with each type of KDT was not the same, so more studies (even multicenter) should be performed to determine the effect of KDT on growth in children with GLUT1DS.

## 5. Conclusions

GLUT1DS should be suspected in children with hypoglycorrhachia in the setting of normoglycemia and low-to-normal cerebrospinal fluid lactate, and refractory epilepsy or movement disorders, especially if they occur during exercise or fasting. In these cases, the analysis of the gene SLC2A1 should be performed and KDT started. Children in whom the genetic study is finally negative, may benefit from treatment. Furthermore, a delay in the treatment could lead to progressive neurological deterioration and they may be treated with ineffective and potentially harmful pharmacological treatments.

KDT for confirmed or suspected GLUT1DS should be initiated as soon as possible. No significant differences were found between different types of KDT. MAD was as effective and safe as CKD in our cohort, so the type of KDT chosen should be individualized. Side effects and deficiencies in vitamins and minerals can appear, but were usually mild an easy to treat in the study, so permanent monitoring is important. KDT should be used life-long in confirmed GLUT1DS. However, the effect of KDT on growth remains controversial.

## Figures and Tables

**Table 1:** Demographic and clinical characteristics of the patients and the ketogenic dietary therapies (KDT) in the study.

	Patients on a KDT (*n* = 18)
Sex	Males, 8 (55.6%) Females, 10 (44.4%)
Median z-score of baseline head circumference (IQR)	−0.5 (−1.9–0.47)
Median age at the onset of symptoms (range)	14 months (4 days to 7 years and 8 months)
Seizure (*n* = 13)	Myoclonic, 5 (41.6%) Typical absences, 3 (25%) Various, 2 (16.6%) Focal onset, 1 (8.3%) Generalized tonic-clonic, 1(8.3%) Clonic, 1 (8.3%)
Movements disorders (*n* = 5)	Paroxysmal exercise-induced dyskinesias, 1 Dystonia and nystagmus, 1 Dystonia and ataxia, 1 Paroxysmal eye movements, 1 Complex movement disorder with a predominance of intentional and postural tremor, 1
Median age at the onset of KDT (range)	5 years and 2 months (3.5 months to 17 years and 4 months)
Age at the onset of KDT	Infant, 2 (11%) 1–2 years, 2 (11%) 2–5 years, 5 (28%) 5–10 years, 6 (33%) >10 years, 3 (17%)
Median time from debut to the onset of KDT (range)	3 year and 1 month (49 days to 13 years and 4 months)
Type of KDT	Classic ketogenic diet (CKD) 3:1, 6 (33.3%) Modified Atkins diet (MAD), 12 (66.7%)
Median time to reach ketosis (range)	3.8 days (2–9 days)
Median length of KDT (IQR)	463 days (170–1863 days)
Reasons for withdrawing KDT (*n* = 10)	Difficulties in compliance, 3 (16.6%) Loss of clinical follow-up, 3 (16.6%) Ineffectiveness, 1 (5.55%) Adverse effects, 1 (5.55%) Length of KDT >2 years, 1 (5.55%) Other, 1 (5.55%)

IQR: interquartile range. KDT: ketogenic dietary therapies.

**Table 2:** Long-term side effects in patients on a ketogenic dietary therapy.

Time on the Diet	3 Months (*n* = 17)	6 Months (*n* = 13)	12 Months (*n* = 10)	24 Months (*n* = 8)
Total of patients suffering side effects	10	5	7	5
Constipation	4	2	1	1
Abdominal pain	-	-	-	1
Nausea/vomiting	1	-	-	-
Anorexia	1	-	-	-
Dehydration	1	-	-	-
Hypoglycemia	1	-	-	-
Hypercholesterolemia ^1^	4	2	3	2
Hypertriglyceridemia ^2^	1	-	1	-
Elevated AST, ALT, or GGT ^3^	1	-	-	-
Hypercalciuria ^4^	3	2	3	1
Hyperuricemia ^5^	-	-	1	3
Metabolic acidosis ^6^	3	-	-	-

^1^ Cholesterol > 200 mg/dl; ^2^ Triglycerides > 150 mg/dl; ^3^ AST: Aspartate aminotransferase > 50 IU/L, ALT: Alanine aminotransferase > 50 IU/L, GGT: Gamma glutamyl transpeptidase > 35 IU/L; ^4^ Urinary calcium/creatinine > 0.2 mg/mg in children ≥ 2 years and > 0.5 in < 2 years; ^5^ Uric acid > 6 mg/dl; ^6^ pH < 7.30.

**Table 3:** Evolution of nutritional markers and micronutrients.

	Lab Reference Values	Mean ± SD				
Nutritional Markers in Blood		Baseline	3 m	6 m	12 m	24 m
Prealbumin (mg/dL)	15–40	17.7 ± 3.5	16.2 ± 3.5	15.7 ± 1.9	15.3 ± 2.9	17.1 ± 3.6
Retinol binding protein (RBP) (mg/dL)	2.5–6.9	3.4 ± 1.2	1.41	1.4 ± 0.07	2.5 ± 0.7	2.5 ± 0.7
Ferritine (ng/mL)	7–140 (<15 y) 15–175 (≥15 y)	31 ± 19	48 ± 31	55 ± 31	52 ± 27	40 ± 21
**Micronutrients**						
Magnesium (mg/dL)	1.5–2.5	2.2 ± 0.28	2.0 ± 0.11	1.9 ± 0.1	2.0 ± 0.08	1.85 ± 0.13
Selenium (µg/L)	70–120	73 ± 8.6	-	76.4	89 ± 6.8	84.4 ± 9.0
Zinc (µg/dL)	70–150	107 ± 4.0	-	97	103 ± 18.8	103 ± 10.3
Total carnitine or Levocarnitine (µmol/L)	21.5–64.5 20–50	52 ± 23 35 ± 2.5	- -	70.5 48.6	61.9 ± 40.3 34.8 ± 6.3	63 ± 25.4 41 ± 13.4
Vitamin A (mg/L)	0.2–0.6	0.31 ± 0.08	-	0.21 ± 0.03	0.28 ± 0.08	0.27 ± 0.07
Vitamin E (mg/L)	3–9 (<12 y) 5–20 (≥12 y)	5.3 ± 1.2	-	3.53	6.0 ± 2.5	5.1 ± 1.4
Folic acid (ng/mL)	3.9–23.9	11 ± 5.2	-	18.1 ± 9.2	20.6 ± 7.8	17 ± 4.6
Vitamin B12 (pg/mL)	250–914	715 ± 545	1023 ± 291	612 ± 9	636 ± 353	859 ± 504
25-hydroxyvitamin D (ng/mL)	20–80	33.3 ± 12.3	-	40 ± 6	36.5 ± 9.2	31 ± 10.0
Calcium (mg/dL)	8.8–10.8	9.6 ± 0.4	9.7 ± 0.4	9.5 ± 0.19	9.6 ± 0.2	9.6 ± 0.2
Phosphorus (mg/dL)	4.5–6.5	5.4 ± 0.9	5.0 ± 0.6	4.8 ± 0.5	4.8 ± 0.7	4.0 ± 0.4

y: years; m: months.

## Data Availability

The data presented in this study are available on request from the corresponding author.
